# Polyarticular Haemophilus Influenza Septic Arthritis in an HIV Patient

**DOI:** 10.7759/cureus.20160

**Published:** 2021-12-04

**Authors:** Taha Faruqi, Adam Green, Daniel P McCall, Matthew Caid, Logan C Smith

**Affiliations:** 1 Orthopaedic Surgery, Beaumont Hospital, Dearborn, USA; 2 Orthopedic Surgery, Beaumont Hospital, Dearborn, USA; 3 Orthopedic Surgery, Beaumont Hospital, Farmington Hills, USA; 4 Orthopaedics, West Virginia School of Osteopathic Medicine, Lewisburg, USA

**Keywords:** arthritis and orthopaedic rheumatology, immunocompromised patient, haemophilus influenza, polyarticular, septic arthrits

## Abstract

*Haemophilus influenzae* is an opportunistic gram-negative bacterium most commonly found in the upper respiratory tract of humans. With the advent of vaccines, most infections caused by these bacteria have been suppressed. However, in the immunocompromised host, an invasive infection may occur, particularly within the musculoskeletal system. In this paper, we present the case of a 55-year-old male with septic shock secondary to polyarticular *Haemophilus influenza* infection. The patient was successfully treated with surgical irrigation and debridement, and antibiotics. Haemophilus infections should be part of the differential in patients with musculoskeletal pain and immunocompromise to avoid potential delays in surgical management.

## Introduction

Haemophilus influenzae (H. influenzae) is a non-motile gram-negative pleomorphic opportunistic bacterium. It is strictly a human pathogen and commonly resides in the upper respiratory tract. There are multiple distinct serotypes of Haemophilus influenza depending on its antigenic polysaccharide capsule [1]. It is most known for causing acute epiglottitis and otitis media, occasionally meningitis, and rarely septic arthritis in nonimmunized immunocompetent pediatric hosts [1]. With the advent of vaccines, there has been a substantial reduction in Haemophilus cases worldwide. However, H. influenzae remains capable of invasive disease in the immunocompromised host. An awareness of the clinical features of H. influenzae infection is necessary to avoid the morbidity associated with delayed or misdiagnosis. In this paper, we present the case of an immunocompromised patient in septic shock as a result of H. influenzae polyarticular joint infection for which surgical irrigation and debridement were performed.

## Case presentation

Our patient was a 55-year-old male with a past medical history significant for human immunodeficiency virus (HIV) who presented to our Emergency Department (ED) with severe shortness of breath, bilateral knee pain, left ankle pain, and left wrist pain. Per the patient, his polyarticular joint pain had been present for two days before his presentation to the ED. Initial vitals: blood pressure 70/36 mmHg, pulse 109 beats per minute, temperature 97.6 F, respiratory rate 20 breaths per minute, and SpO2 98%. Initial lactic acid (LA) was 5.9 mmol/L, erythrocyte sedimentation rate (ESR) was 108 mm/hr, and C-Reactive Protein (CRP) was 280 mg/L. A 30 ml/kg 0.9 normal saline (NS) fluid bolus was initiated, and a repeat LA two hours later was 3.4 mmol/L. Serum white blood cell count (WBC) was 2.8x10^9^/L, hemoglobin was 14.1 g/dl, and platelet count was 41x10^9^/L. Vancomycin, Ceftriaxone, and Piperacillin-Tazobactam were initiated, and blood cultures were obtained. Absolute CD4 count was 40 cells/mm^3^ (last recorded CD4 from 9 months prior was 663 cells/mm^3^). Per chart review, the patient had previously followed with an infectious disease specialist and was currently on highly active antiretroviral therapy due to the significant drop in CD4 count. The patient remained on antiretroviral medications during admission by the Infectious Disease (ID) service.

Orthopedic surgery and rheumatology were consulted for acute polyarticular pain. An effusion was present in his bilateral knees and right ankle, which was appreciable on physical exam and confirmed with imaging studies. His left ankle and left wrist joints were painful but did not have significant effusions upon a range of motion. Bilateral knee and ankle joints were aspirated, although no aspirate was obtained from the left ankle. Cell count revealed a WBC count of 119,625 cells per microliter from the left knee and 128,640 cells per microliter from the right knee. Given his symptomatology, a decision was made to perform formal irrigation and debridement of his bilateral knees, bilateral ankles, and left wrist. Intraoperatively, purulent discharge was encountered in all five joints, and cultures were obtained at that time. Preoperative aspiration cultures from the right knee and right ankle grew H. influenzae, beta-lactamase negative; however, the intraoperative culture specimen was negative on all final cultures. The patient was subsequently placed on ceftriaxone. Post-operatively, the patient continued to improve. His CD4 count increased to 741 cells/mm^3^. Ceftriaxone was discontinued by the ID service on postoperative day 18, after which he was discharged home.

## Discussion

H. influenzae is a well-known microorganism causing sinusitis, pneumonia, and epiglottitis. With the widespread use of the Hib-conjugated vaccine, the rate of most of these infections has decreased substantially [2]. Less common but more severe infections associated with H. influenzae include meningitis and septic arthritis, particularly in the pediatric population. However, in the adult population, less than 1% of septic arthritis cases are caused by H. influenzae [3]. When present, H. influenzae septic arthritis commonly occurs in patients with serious underlying medical comorbidities or an immunocompromised state.

Septic arthritis in the immunocompromised host is a challenging diagnosis. The classic presentation of septic arthritis of a warm, tender, swollen, erythematous joint and inability to bear weight may not be present on initial examination. Thus, clinical suspicion must remain high at the time of evaluation in any patient with joint pain with a history of immunodeficiency. In the case of immunocompromise, septic arthritis may be associated with typical microorganisms and opportunistic microorganisms [4,5]. Standard treatment involves surgical debridement and culture of fluid and tissues with empiric antibiotic treatment based on the most likely causative organism [3]. Our patient was immunocompromised secondary to HIV infection and presented with sepsis from widespread musculoskeletal Haemophilus infection. Due to the limited report of such cases and infrequency of polyarticular involvement, we felt that a discussion of appropriate evaluation and treatment would benefit those treating joint infections.

Previous reports have identified H. influenzae as a cause of septic arthritis in the immunocompromised and immunocompetent host with polyarticular involvement. In those cases, polyarticular involvement was associated with a previous history of multiple myeloma, splenectomy, alcoholic liver disease, rheumatism, chronic steroid use, and laryngeal cancer [4-8]. Our case is similar to previous reports in that our patient was severely immunocompromised, as evidenced by the low CD4 T-cell count. Our case differs from previous reports of polyarticular septic arthritis caused by H. influenzae in that our patient is co-infection with HIV disease. In all cases, patients were successfully treated with aspiration of the joint and IV antibiotics with or without formal debridement (table 1).

**Table 1:** Clinical features of previously reported adult cases of Haemophilus influenzae septic arthritis including our case M- male; F- female; Rt- right; Lt- left; I&D- Irrigation and Debridement; CVID- Common Variable Immunodeficiency.

Ref	Age	Gender	Predisposing Condition(s)		Shock	Location	Treatment		Outcome
Our case	55	M	Human Immunodeficiency Virus (HIV)		Yes	Bilateral knee Lt ankle Lt wrist		I&D	Resolved
Hawkins et al [4]	35	M	Common Variable Immunodeficiency (CVID)			Lt knee		Aspiration Aztreonam	Resolved
Turner et al [5]	57	M	Laryngeal cancer Gout Alcoholism		No	Rt knee Lt wrist		I&D Ampicillin	Resolved
Lester et al [6]	40	M	Alcoholic liver disease		Yes	Bilateral knee Bilateral ankle Lt elbow Lt wrist Lt knee		Aspiration Cefuroxime	Resolved
Saba et al [7]	46	F	Multiple myeloma		Yes	Bilateral knee Rt ankle Rt elbow Lt wrist		Aspiration Ampicillin	Resolved
Melhus and Svernell [8]	46	F		Rheumatoid arthritis Chronic steroid use		Rt shoulder Rt elbow Rt knee Lt wrist Lt hand		Aspiration Ciprofloxacin	Resolved

## Conclusions

Musculoskeletal infections caused by H. influenzae are rare in the adult population. Intra-articular infection requires early diagnosis and prompt operative intervention to mitigate effects caused by bacteria and the ensuing inflammatory cascade, which can be exacerbated by underlying immunodeficiencies (i.e., HIV). Our case presents an HIV-positive patient with septic shock as a result of polyarticular Haemophilus influenzae infection. In addition to the ID service continuing appropriate retroviral medication, our patient was successfully treated with antibiotics, irrigation, and debridement.
